# The Potential of Using Chitosan on Cereal Crops in the Face of Climate Change

**DOI:** 10.3390/plants10061160

**Published:** 2021-06-07

**Authors:** Joanna Kocięcka, Daniel Liberacki

**Affiliations:** Department of Land Improvement, Environmental Development and Spatial Management, Poznań University of Life Sciences, Piątkowska 94, 60-649 Poznań, Poland; daniel.liberacki@up.poznan.pl

**Keywords:** chitosan, chitooligosaccharides, nanoparticles, maize, wheat, rice, millets, barley, cereal

## Abstract

This review presents the main findings from measurements carried out on cereals using chitosan, its derivatives, and nanoparticles. Research into the use of chitosan in agriculture is growing in popularity. Since 2000, 188 original scientific articles indexed in Web of Science, Scopus, and Google Scholar databases have been published on this topic. These have focused mainly on wheat (34.3%), maize (26.3%), and rice (24.2%). It was shown that research on other cereals such as millets and sorghum is scarce and should be expanded to better understand the impact of chitosan use. This review demonstrates that this chitosan is highly effective against the most dangerous diseases and pathogens for cereals. Furthermore, it also contributes to improving yield and chlorophyll content, as well as some plant growth parameters. Additionally, it induces excellent resistance to drought, salt, and low temperature stress and reduces their negative impact on cereals. However, further studies are needed to demonstrate the full field efficacy of chitosan.

## 1. Introduction

Cereals are one of the most important crops on Earth. They are responsible for feeding the human population of the world. Cereal grains account for more than half of the world’s daily caloric intake [1]. It is therefore not surprising that cereals account for as much as half of the global cultivated area [2]. Nowadays, the biggest challenge for cultivation is climate change. Increased CO_2_ concentrations, extreme weather phenomena, rising temperatures, and droughts can be stress factors for many crops. Consequently, climate change also directly affects cereal production. One of the factors reducing yields is air temperature increase. It has been shown that global wheat production decreases by 6% for every 1 °C increase in air temperature [3]. Researchers emphasize that plant tolerance to higher temperatures may be a key trait for wheat yields in the future. Furthermore, it is extremely important to achieve drought tolerance [4,5]. This is particularly important for water-limited areas. It should be noted here that the production of and demand for cereals is constantly increasing, while the water resources required for cultivation are not rising. Another problem is the constantly increasing demand for water, for purposes other than agriculture. This phenomenon will be the main reason for water shortages for agriculture in the coming decades in China [6]. It is therefore extremely important to take actions in agriculture to help plants survive and adapt to climate change to guarantee food security. There are various methods to reduce the impact of climate change on plant physiology and the yield losses associated with its deterioration. These include improved irrigation, appropriate use of fertilizers, and increased emphasis on breeding varieties that will be more resistant to these changes [7]. Furthermore, another solution is to develop varieties with superior genetic yield potential and stress adaptation [8]. It may also be beneficial to use special antitranspirant formulations to allow cereals to better use and accumulate water to survive a drought period. Antitranspirants are chemicals that reduce transpiration rate. Limiting transpiration through their use on plants is beneficial in the context of increasing demand for limited water resources. The application of antitranspirants is a great opportunity for the proper development especially of those crops that require irrigation [9]. One of the compounds included in the broad group of antitranspirants is chitosan.

Chitosan is a derivative of chitin and is considered the second most common polymer in the world after cellulose [10]. Chitosan, as well as chitin, are classified as polysaccharides containing randomly distributed β-(1-4)-linked D-glucosamine and *N*-acetylglucosamine units. Chitosan is mainly obtained from leftovers obtained from seafood processing such as crab and shrimp shells, as well as fish scales. Furthermore, the potential to receive it from waste fungal mycelium is also indicated [11,12]. In agriculture, chitosan is used through foliar application to plants, seed treatment, or as a direct soil fertilizer. 

During studies involving the use of this agent on plants, its antitranspirant properties have been repeatedly confirmed. Research on peppers has shown that chitosan decreases transpiration through partial or full closure of stomata [9]. Reduction of transpiration by the application of chitosan was also observed in the case of *Phaseolus vulgaris* L. [13] and after spraying coffee leaves through chitosan oligomers [14]. Moreover, chitosan has been identified as a growth promoter [15,16,17]. Furthermore, this product exhibits properties helping to control diseases and prevent crop pathogens. It affects the elicitation of defense-related enzymes [18]. It also has fungicidal or fungistatic potential, which is confirmed by the effect of chitosan on the morphology of microorganisms [19]. Due to its high efficacy in disease control, and the fact that it is biodegradable and environmentally friendly, it ought to perform well, not only in conventional farming but also in organic farming [20]. Worldwide research is increasingly considering the use of chitosan derivatives, besides chitosan alone, as well as nanoparticles. Moreover, the combination of chitosan nanoparticles with other elements such as copper (Cu), zinc (Zn), selenium (Se), as well as silver (Ag) is becoming widely used [21,22,23]. Researchers suggest that chitosan based metallic nanoparticles may be even more effective than bulk chitosan in anti-pathogenic and plant growth-promoting activities [24]. Also in the research, the potential for the use of chitosan and its derivatives in agriculture and the need to systematize the current state of knowledge is repeatedly highlighted. This manuscript aims to review published research findings on the impact of chitosan on cereal production. Moreover, it focuses on the role of chitosan as a management tool. This paper presents how the application of this product affects plant parameters and indices. Consolidating knowledge in this area will allow a comprehensive presentation of the role of chitosan in cereal cultivation. 

## 2. Methodological Framework

The aim of this review is to present, systematize and evaluate the current state of knowledge concerning the effects of chitosan on the most important plant group in the world, namely cereals. In the article, significant plant parameters and traits influenced by chitosan were selected. These are presented in subsections such as yields, growth parameters, chlorophyll content, gas exchange, water-use efficiency (WUE), relative water content (RWC), pathogen and disease control. This paper presents the results of research conducted since 2000 with their synthesis and evaluation. Original scientific articles registered in Web of Science, Scopus, and also Google Scholar were taken into consideration for this analysis. The search included the phrase: chitosan AND the name of the cereal. Cereals were defined as wheat, rice, maize, barley, oats, rye, sorghum, and millets. For this review, studies involving chitosan derivatives and nanoparticles were also eligible. To synthesize the current state of research, one of the last chapters presents the number of articles found and current research trends in the application of chitosan on cereals. The number of papers specified in this review is the final score obtained after filtering the search results in the Web of Science, Scopus and Google Scholar databases and excluding articles that did not match the research topic.

## 3. Effects of Chitosan on Cereal Yields

The parameters that are of most interest from the point of view of grain production are yield and its indicators. Regardless of its other positive properties, chitosan has no chance of becoming widely used on cereals if it does not have a positive effect on yield. Many studies have been carried out in this context. In the case of wheat, the application of chitosan resulted in a 13.6% increase in yield per hectare in comparison with a control crop. Furthermore, it had a positive effect on tillers per plant, spikes per plant, and 1000 grain weight [25]. It was also shown that exogenous use of chitosan on this plant improves other yield parameters such as a number of grain/spikes, grain index as well as grain yield. Their increase was also recorded under moderate and severe drought conditions [26]. Moreover, under reduced irrigation conditions, a beneficial effect of chitosan was observed concerning economical yield. It was also noted that the use of chitosan together with hydrogel enhances this result [27]. For wheat, the application of chitosan under limited irrigation conditions slightly improved the biological yield and grain yield, and improved it significantly for 1000 grain weight. The result for plants without stress could not be reached [28]. Moreover, research on maize has shown that the application of chitosan under low-temperature conditions increases the germination index. Furthermore, it also reduces the average germination time [29]. Similar positive results were obtained in a study on barley (*Hordeum vulgare* L.) under semi-arid conditions. A significant improvement of grain yield per plant, number of spikes per plant and number of grains per spike, and 100-grain weight compared to samples without chitosan was observed [30]. Also after the application of chitosan with DAP fertilizer (Diammonium phosphate 46% P2O5), an increase of the same parameters was achieved [31]. Another cereal on which research was conducted was maize. Tests on this plant also demonstrated that an enhanced grain yield was obtained after the use of chitosan [32]. Moreover, measurements carried out on this cereal after application of chitosan and its derivatives: N-succinyl chitosan and N, O-dicarboxymethylated chitosan (each separately and a mixture of two together) showed that all these substances increased grain yield and harvest index. However, it was the highest for the mixture of derivatives [33]. Studies have also been carried out on the effects of the use of chitosan with plant growth promoting rhizobacteria (PGPR). It was found that this combination can improve maize production [34]. Application of Cu-chitosan nanoparticles significantly increased grain yield and 100 grain weight of maize [35]. A positive effect on grain yield was also observed after using chitosan-silicon nano-fertilizer on maize (*Zea mays* L.) [36]. In the case of Zn-chitosan nanoparticles application on maize, an increase in grain yield from 20.5 to 39.8% was achieved [37]. Studies carried out on wheat also showed that the foliar application of nano chitosan nitrogen, phosphorus, and potassium fertilizer enhances yields [38]. The harvest index as well as the crop index also improved after this treatment compared to the control sample [39]. Additionally, research has been conductedinto the use of chitosan oligosaccharides on wheat. They showed that the tillering stage and returning-green stage of plants were the most sensitive to this application. It was also proven that treatment with chitosan oligosaccharides has the potential to increase yield components, such as spike number and grains per spike [40]. Moreover, the multifunctional complex ’Vitaplan CL + 0.1% Chitosan’ was also applied in wheat experiments. After its use, yields were increased by 24.4% compared with the control [41]. Furthermore, other multifunctional biologics with chitosan have also been shown to positively affect grain yield and quality [42]. The research was also conducted under salinity stress conditions on two wheat varieties. It was found that plants treated with chitosan had better results regarding 1000 grain weight, straw yield, and biological yield as well as grain yield [43]. Furthermore, a positive effect of chitosan nanoparticles as well as chitosan nanoparticles loaded with *N*-acetylcysteine under ozone stress conditions, was also observed. This treatment was shown to increase the weight of 1000 durum wheat seeds [44]. In studies on the application of nanochitosan particles to finger millet, an improvementin yields was noted compared to the control sample [45]. This is in agreement with subsequent research, where an increase in grain weight as well as grain yield, was obtained. However, no significant effect was noticed on the number of inflorescences [46].

Studies on cereal treatment with chitosan have considered not only different varieties of this antitranspirant, but also various concentrations and application methods. One such study was the application of varying concentrations (0, 50, 75, 100, and 125 ppm) of chitosan on maize in the early stages of growth. It was found that the greatest seed yields are obtained at the two highest concentrations [47]. Measurements were also conducted concerning the application of chitosan with different molecular weights (monomer, oligomer, polymer) and three different methods of application (seed soaking, seed soaking + foliar spraying, foliar spraying). The potential to obtain higher yields was found after soaking the seeds before planting rice and then spraying foliar polymeric chitosan four times in a dose of 20 ppm [48]. Later measurements of the application of different variants of chitosan on rice confirmed that the maximum yield was achieved after seed soaking and soil application. The authors indicate that this is because, in soil, chitosan is available to plants longer than in foliar spray. An increase in seed numbers per panicle was also noted, but it was not significant [49]. Studies carried out on rice plants in Vietnam also point to promising yield increases after chitosan [50]. The use of chitosan nanoparticles on wheat increased grain yield. Interestingly, foliar application had a better effect than applying chitosan to the soil. This is not fully consistent with the results described above. An increase in biomass, spike weight, number of grains per spike, and grain yield was also noted. According to the authors, this improvement is due to several factors such as changes in transpiration and improvements in the rate of photosynthesis and water status in wheat. Also, modifications to the leaf organelle ultrastructure and the maintenance of the nutrient supply have an impact [51]. Moreover, the application of chitosan nanoparticles on barley (*Hordeum vulgare* L.) resulted in an improvement of the number of grains per spike, the grain yield as well as the harvest index. Additionally, the highest 1000-grain weight value was obtained with the highest nanoparticle dose of 90 ppm [52].

From the above review, it is clear that chitosan has a strong effect on improving cereal yield. Furthermore, it is effective both as chitosan itself and as its derivatives and nanoparticles. A trend towards more beneficial results at higher concentrations is noticeable. However, no clear conclusions can be drawn as to which application method is the most effective. Certainly, chitosan has a positive effect on yield and yield indices regardless of the type of grain. The conclusions of the above review are based on a large number of field studies [25,26,27,28,30,31,38,40,42,43,44,47,50,51]. Moreover, they have also been verified by experiments conducted under laboratory [36,37], and greenhouse conditions [33,34,35,45,48]. Furthermore, this review has shown that, chitosan has the potential to reduce the effects of drought stress, salt stress, and low temperatures, thus improving the yield. The widespread use of chitosan on cereals, especially in regions of the world particularly exposed to the negative effects of various types of stressors, would make it possible to mitigate the yield loss caused by environmental changes. It should be noted that studies have shown that chitosan can cope with a wide range of conditions. This indicates the potential of chitosan as a universal tool that can be used worldwide, regardless of climate to improve yields.

## 4. Growth Parameters

Besides yield, growth parameters are also an important aspect from the point of view of global grain cultivation. More than half of the articles considered in this review took into account the effect of chitosan on this group of parameters. For a clearer presentation, it has been decided to divide this chapter into subchapters on different plants.

### 4.1. Wheat and Barley

One of the experiments conducted was the application of chitooligomers (COS) on wheat. An increase in root fresh weight and also in shoot fresh weight of wheat (*Triticum aestivum* L. cv. Jimai 22) was observed. Depending on the type of COS, an increase in root fresh weight of 21.8% to as much as 53.2%, compared to the control sample, was achieved [53]. Also, tests on wheat have shown that chitosan hexamers with different degrees of acetylation improve growth parameters [54]. Moreover, research has been carried out on a water-soluble form of chitosan, namely chitosan hydrochloride. It has been shown that this substance stimulates growth and affects germination rate and root development of durum wheat [55]. Comparative studies were also conducted on the effect of different concentrations of chitosan as well as chitosan nanoparticles on wheat. It was shown that the best growth effects occur at 50 μg/mL for chitosan and 5 μg/mL in the case of nanoparticles. This includes parameters such as seedling length, number of adventitious roots, seed germination percentage, germination index, vitality index, fresh weight, the ratio of root/shoot and seedling index. A better effect has been shown with a lower concentration of nanoparticles than chitosan. This is due to higher adsorption on the surface of the wheat seeds [56]. Moreover, the studies undertaken on wheat also showed an increase in root length, shoot length, fresh weight, dry weight after foliar application of chitosan nanoparticles loaded with nitrogen, phosphorus and potassium [39]. In a study of the use of chitosan nanoparticles on barley (*Hordeum vulgare* cv. Reyhan) and wheat (*Triticum aestivum* cv. Pishtaz), two application methods of seed priming and direct exposure were also compared. Positive effects on seedling, root and shoot lengths were observed, with seeds priming but only at low chitosan nanoparticle concentrations of 30 ppm. At high values like 90 ppm this treatment had negative effects on growth characteristics in both application methods [57]. In barley studies, the application of 1, 3, and 6 g/L of chitosan resulted in a significant improvement in plant height, but not in spike length [30].

Based on research over the last 20 years, it can be concluded that in the case of wheat, chitosan has a positive effect on growth parameters. This concerns both germination and root parameters. Nanoparticles, especially in low concentrations, also contribute to the improvement of many parameters. The number of publications on barley is not as high as for wheat. However, in papers the positive effect of chitosan on growth parameters has been noted a few times also for barley. 

### 4.2. Millets

Another cereal type analysed in the context of chitosan application was millet. Tests carried out using an Elexa preparation containing chitosan for pearl millet demonstrated improvements of plant height at the vegetative phase, number of tillers, and 1000 seed weight [58]. A positive effect was also achieved in subsequent studies using chitosan nanoparticles, increasing seed germination percentage and seedling vigor [59]. Also, in the case of finger millet, the application of chitosan nanoparticles was shown to increase the number of leaves, leaf length, and shoot length. Furthermore, an improvement in dry weight of up to 52% compared with the control sample was observed [46]. In other studies on the use of nano-chitosan particles on this same plant, no major influence on the means of leaf length and number was found. However, a significant impact on dry weight was observed [45]. Also, the effect of the copper-chitosan nanoparticle (CuChNp) in two different methods of application, as a foliar spray as well as combined application (involving seed coat and foliar spray), on finger millet (*Eleusine coracana* Gaertn.) was studied. In both treatments, an increase in several parameters such as the number of leaves, leaf length, shoot height, as well as fresh and wet plant weight, was observed. The highest increase was recorded in the case of dry weight, where it reached 297% in the combined application and 152% in the foliar treatment [60]. Despite the relatively small number of studies in relation to other cereals, measurements conducted on millets show that chitosan nanoparticles contribute to the improvement of many growth parameters. Nevertheless, more research is desirable, to strengthen the hypothesis of the favourable impact on this cereal’s growth indices. These results could be extremely valuable, considering that this cereal is a source of food in many poor countries.

### 4.3. Maize

Research has also been conducted on maize. It was shown that the use of chitosan in the early stages of development improved plant height, leaf number, leaf length and breadth, and leaf area [47]. In addition to improving plant height and leaf surface, the effect of the substance on the growth of shoot and root dry weight has also been reported [61]. Furthermore, in the case of maize, chitosan improved root length, root surface, and root volume also under cadmium stress conditions [62]. Positive results were also obtained in later studies with foliar application of different concentrations of chitosan nano-fertilizer (0.01, 0.04, 0.08, 0.12, and 0.16%) on the same plant. Improvement of parameters such as plant height, root length, root number, and stem diameter was shown. The highest values were observed at a concentration of 0.16% [63]. It was also found that the application of an adequate dose of chitosan-based nanoparticle fertilizer increased the fresh and dry biomass of maize [64]. Moreover, research has been conducted using a nutrient-water carrier prepared with N, O-carboxymethyl chitosan, among others. Its application had a positive effect on the root length and seedling height of maize [65]. Also after the use of nanochitosan on this plant, a growth-promoting effect on plant height and leaf area was observed [66]. Other studies on rice have also confirmed that chitosan nanoparticles (ChNP) affect growth parameters. This treatment resulted in a higher number of leaves and plant length compared to the control sample [67]. It also confirmed that the combination of moringa and chitosan had a beneficial effect on the index-seed germination and growth parameters of maize [68]. Furthermore, it has been proven that coating the seeds with chitosan increases not only the height of maize plants but also of sorghum. An increase in seed germination was also found for these crops [69]. 

However, there are also some articles with opposite results [70]. In one of them, no effect on plant height, root length, leaf surface, shoot and root area and total dry matter was observed 10 days after chitosan application to maize. Further studies have reported a positive reaction of chitosan on maize hybrids (DKB 390 and DKB 390 VTPRO) increasing the percentage of germination. There was no impact on root length, nevertheless, its anatomy changed [71]. Other measurements show that a chitosan coating on maize did not affect the rate of seed emergence in comparison with the control sample. Moreover, it did not contribute to higher seedling germination rates [72]. However, these are not the only studies showing a reduction in growth parameters. Other measurements showed negative effects of chitosan on maize (*Zea mays* L.) seed emergence. This result may be due to the high concentration of the polymer applied and the simultaneous use of fertilizers and salicylic acid [73]. Moreover, when using high concentrations of polymeric chitosan/tripolyphosphate on maize, germination inhibition was reported [74]. Therefore, the results obtained for chitosan applied on maize are contradictory.

Research involving the use of Cu-chitosan nanoparticles on maize seedlings showed an increase in germination percent, shoot and root length, root number, seedling length, fresh and dry weight, and seed vigor index, but only at concentrations of 0.01 to 0.12%. At a concentration of 0.16%, inhibition of seedling growth was observed. The authors explain that this is due to excessive Cu content, and thus a decrease in metabolic enzymes [75]. The growth of plant height, stem diameter, root length, and root number after application of Cu-chitosan nanoparticle on maize at concentrations of 0.01 to 0.12% was also observed in later studies [35]. The positive effect of this treatment was also noted for shoot length, fresh as well as dry weight [76]. The research was also carried out on seed treatment of maize with salicylic acid-chitosan nanoparticles (SA-CS NPs). These showed an enhancement in germination percent, shoot-root length, fresh weight, and seedling vigor index with this use. Interestingly SA-CS NPs showed much better results than bulk chitosan alone [77]. A positive effect was also demonstrated for Zn-chitosan nanoparticles. It has been proven that its use increases maize parameters, such as plant height, stem diameter, as well as root length [37]. Studies were also conducted on the application of chitosan-silicon nano-fertilizer on maize (*Zea mays* L.). They showed a significant increase in leaf area, shoot length, root length as well as root number compared to an untreated control sample [36].

On the basis of the review carried out, it can be concluded that the results with regard to maize are not conclusive. There are many studies showing that chitosan or chitosan varieties have a positive effect on growth parameters, in particular plant height and root characteristics. However, there are also several articles that show negative or no influence of this substances on growth parameters. It can therefore be concluded that more research should be carried out on maize, with close monitoring of the experimental conditions. In this way, it will be possible to analyse them in detail in relation to the individual factors influencing the results.

### 4.4. Rice

In relation to growth parameters, a number of studies have also been carried out on rice (*Oryza sativa* L.). In one of these, no effect of chitosan on seedling growth was observed. The authors believe, the concentration of the substance may justify this result [78]. Measurements were also carried out to cover the application of chitosan with different molecular weights (monomer, oligomer, polymer) and three different methods of application (seed soaking, seed soaking + foil spraying, foil spray). No significant changes in the height of rice plants were noticed. However, it has been shown that the application of the polymer by seed soaking before planting and then four foliar sprayings with chitosan significantly increases dry matter accumulation. Therefore, it was concluded that this form of application stimulates growth [48,49]. The increase in dry matter is also confirmed by subsequent studies on the use of chitosan (both oligomeric and polymeric). Moreover, an improvement in plant height, leaf, and fresh root weight was also obtained. Interestingly, the best results were obtained for oligomer with an 80% degree of deacetylation at 40 mg/L [79]. Also, the application of oligochitosan was shown to enhance rice growth [80]. Other studies showed that chitosan oligosaccharides stimulated the growth of rice roots and stems. Furthermore, it stimulated metabolic processes and photosynthesis at the seedling stage [81]. Research has also been conducted into the use of lanthanum-modified chitosan oligosaccharide nanoparticles (Cos-La) on rice (*Oryza sativa* L). It has been shown that Cos-La enhances plant height and fresh weight [82]. 

As we can see, the results concerning the influence of chitosan, and also its varieties and nanoparticles on cereal growth parameters are not unequivocal. Many studies report improvements for each of the cereals considered. However, there are also some reports emphasizing no effect, or even a negative effect of chitosan, specifically for maize. Researchers emphasize that the differences obtained in plant reactions (concerning growth parameters) to chitosan application may result from the physicochemical properties of chitosan (deacetylation degree, molecular weight, and viscosity), as well as plant type [79,83]. Moreover, it is suggested that various seed germination reactions with chitosan may result from the concentration of the substance used, the experimental conditions, and the specific seed and crop features [72]. To understand the exact influence of chitosan, its derivatives, and nanoparticles on growth parameters, a more detailed analysis is needed. Such an analysis would consider each parameter separately, the type of application, its concentration, and individual factors during the experiment. This topic could be an interesting development of this review.

### 4.5. Different Stress Conditions

Studies were also carried out on the use of chitosan under different types of stress (e.g., drought, salt, temperature). It was decided to describe their results, irrespective of the type of cereal, in a separate section, as they are a valuable source of knowledge in the face of climate change. 

Under drought conditions, chitosan also promoted improvements in fresh and dry root weight. Furthermore, it was shown that lower concentrations of chitosan had a higher efficacy [84]. Also, studies under these conditions on barley indicated that chitosan improved plant height, number of leaves, and leaf area [85]. Exogenous application of chitosan on wheat was proven to have a positive effect on plant growth parameters. As a result, an increase in flag leaf area and also shoot dry weight was observed. Moreover, the improvement of these parameters was also observed under conditions of limited irrigation [26]. This indicates the potential of using chitosan during drought conditions. Measurements were carried out in rice (*Oryza sativa* L. ‘Leung Pratew123’) exposed to osmotic stress. After its rehydration, crops treated with chitosan showed higher fresh and dry weight [86]. Furthermore, chitosan showed a positive influence on leaf area and biomass in rice also under difficult conditions of elevated ozone concentration [87]. A positive effect of chitooligomers (COS) with different degrees of polymerization on wheat (*Triticum aestivum* L. Jimai 22) under chilling stress was also observed. The results obtained indicate that this treatment improved growth parameters such as shoot and root length as well as fresh and dry weight under stress conditions [88]. Similar results were obtained in maize where a significant increase in shoot height, dry weight, root dry was recorded under low temperature conditions [29]. Also under cold stress conditions, chitooligosaccharide (COS) improved fresh root and shoot weight, as well as rice root vigor [89]. Thus, it can be concluded that chitosan has potential not only in plant adaptation to drought, but also to cold. Improvements in growth parameters were also noted after the use of chitooligosaccharide and also sulfated chitooligosaccharide (SCOS) under salt stress conditions. An increase in shoot length, root length, wet weight, and dry weight of wheat (*Triticum aestivum* L. Jimai 22) was observed [90]. Improvement of growth parameters under salt stress conditions was also noticed when chitosan was used on the varieties Sakha 94 and Gemmieza 9 [43]. In the case of maize, a reduction in the negative effects of salt stress on shoot dry weight was also observed after chitosan application [61]. Furthermore, maize treated with chitosan under this stress condition showed an improvement in parameters such as root length and plant height [91].

All of these results indicate that chitosan does an excellent job of mitigating the effects of stress cereal growth parameters. It had beneficial effects under drought, cold, salt stress, and elevated ozone concentration conditions. This is particularly important, given that many areas of the world are currently facing increasing stress phenomena. The results of this review highlight the considerable potential of chitosan for cereal adaptation to climate and environmental change.

## 5. Chlorophyll Content

In worldwide research, chlorophyll a, chlorophyll b, as well as total chlorophyll are among the most frequently determined content of photosynthetic pigments. Chlorophyll is responsible for the green color of plants. Moreover, chlorophyll content has also been shown to be an indicator of photosynthetic capacity [92]. Further, it can be used to better predict biomass and crop productivity [93]. Also, a relationship between midday gross primary production (GPP) and total crop chlorophyll content has been discovered [94]. In measurements involving the application of chitosan to cereals, parameters determining chlorophyll have not been ignored. An attempt has been made to determine how this product affects the chlorophyll content of plants.

Studies carried out on wheat showed that chitosan improved chlorophyll content. This had a positive effect on plant growth under drought stress [25]. Also, research conducted under moderate and severe drought conditions showed that wheat treated with chitosan achieved higher values of total chlorophyll as well as total carotenoid concentration [26]. In studies on rice under drought conditions, an improvement in chlorophyll a and b were obtained for plants with chitosan [84]. Moreover, after the application of chitosan at concentrations of 0.01 to 0.12%, an increase in chlorophyll a and b content was recorded in maize [35]. Similar results were obtained in subsequent studies using different concentrations of chitosan nano-fertilizer (0.01, 0.04, 0.08, 0.12, and 0.16%) on the same plant. In all cases, the substance caused an increase in total chlorophyll with the highest concentration of 0.04% [63]. It has also been shown that the application of chitosan has a positive effect on increasing the greenness of maize leaves [95]. On the other hand, in the case of rice, no significant changes were observed in leaf greenness as measured by chlorophyll meter [49]. Other measurements carried out on rice (*Oryza sativa* L. ‘Leung Pratew123’) immediately before exposure to osmotic stress, noted an increase in Chl a content. An increase in chlorophyll b and carotenoids was also observed, but was not significant. After osmotic stress, the results were variable. The cultivar LPT123 maintains elevated pigment levels but its salt tolerant mutant line (LPT123-TC171) does not [86]. In the case of wheat (*Triticum aestivum* L. Jimai 22), treatment with chitooligosaccharide and also with sulfated chitooligosaccharide improved chlorophyll a and b content under salt stress conditions [90]. An increase in photosynthetic pigments was also recorded in two other wheat varieties Sakha 94 and Gemmieza 9 under salt stress. Chitosan-treated plants recorded higher chlorophyll a, chlorophyll b, as well as carotenoids, compared to the control [43]. Studies on rice noted a slight increase in chlorophyll b/a, which has the potential to maximize yields. Also, this research suggests that chloroplast is a target organelle for chitosan action [79]. Furthermore, chitosan reduced the negative effect of elevated ozone concentrations on chlorophyll in the case of rice. After 7 days of ozone exposure, the chlorophyll values of plants treated with chitosan were similar to the unstressed control [87].

In addition to chitosan, studies have also been carried out using its derivatives and nanoparticles. The chlorophyll aspect was not omitted in these either. The impact of chitosan and its derivatives (*N*-succinylchitosan and N, O-dicarboxymethylated chitosan) on maize under water stress conditions was also analysed. The results indicate that the above treatments affect photosystem II activity. Moreover, after the application of these substances, the content of chlorophyll, a, and a+b was higher in comparison with the control sample also subjected to water stress [96]. An increase in chlorophyll content was also observed after the application of chitosan nanoparticles on wheat. The highest result was obtained, with the highest tested substance concentration of 90 ppm [51]. Other studies on wheat have shown that 5 μg/mL chitosan nanoparticles have a positive effect on chlorophyll and photosynthetic capacity [56]. After the application of copper-chitosan nanoparticles (CuChNp) on finger millet, the chlorophyll content increased. Foliar application increased it by 32%, and combined application (involving seed coat and foliar spray) by 84% [60]. An increase in chlorophyll content was also demonstrated after the application of Zn-chitosan nanoparticles on maize [37]. Treatment of maize seeds with salicylic acid-chitosan nanoparticles (SA-CS NPs) also improved total chlorophyll [77]. Studies have also been conducted on the effect of chitooligomers (COS) with different degrees of polymerization on wheat (*Triticum aestivum* L. Jimai 22) under chill stress. COS was shown to mitigate the stress-induced decrease in chlorophyll content [88]. Furthermore, also under water stress conditions, barley treated with chitosan showed an increase in chlorophyll a and b [85]. The effect of increasing total chlorophyll content was also found during tests using Chitosan-silicon nano-fertilizer on maize (*Zea mays* L.) [36]. Studies were also conducted using chitosan nanoparticles containing the NO donor S-nitroso-mercaptosuccinic acid. This agent applied to maize was shown to prevent salt-induced changes in chlorophyll content and maximum quantum yield of photosystem II [97]. 

In general, in studies involving both the use of chitosan and its derivatives or nanoparticles, it is possible to observe a beneficial effect on chlorophyll. The impact of antitranspirant on this parameter was investigated under both field [25,26,43] and pot conditions [49,63,84]. The results of scientific research show that, regardless of the type of cereal, chitosan increases chlorophyll content. Furthermore, chitosan also copes well with drought and salt stress, as well as low temperatures. These results are particularly promising in the context of ongoing climate change. Perhaps chitosan will be an effective tool for maintaining, and even improving chlorophyll content regardless of adverse plant conditions.

## 6. Gas Exchange

This chapter considers the parameters directly related to gas exchange. These include photosynthetic rate (P_N_), stomatal conductance (gs), and intercellular CO_2_ concentration (Ci). The effects of chitosan on all of these components have been reviewed. An experiment carried out on maize showed that the net photosynthetic rate (P_N_) decreased on the first day after chitosan application. However, on the following days, it increased, but only on the third day was it statistically significant. Then P_N_ was higher than in the control fields by 10 and 18% depending on CH5 pentamer concentrations (10^−5^ and 10^−7^ M) [70]. Similar results were obtained in subsequent studies, where not only chitosan but also its derivatives: N-succinyl chitosan and N, O-dicarboxymethylated chitosan were used on maize. Research conducted under water scarcity conditions has shown that on the first day of stress the photosynthetic rate (P_N_) in both the treatment of chitosan and derivatives decreased. After 15 days of the experiment, P_N_ increased, while after rehydration, it reached the value of a non-stress (irrigated) control sample [96]. This was also confirmed by later tests, where after 15 days the chitosan derivatives (each separately), and also their mix, contributed to an increase in the photosynthetic rate [33]. Pearl millet also showed a decrease in photosynthesis rate under drought stress. Initially, it was even greater in chitosan-treated plants than in control plants. However, after 7 days, plants with chitosan had a much higher photosynthetic rate [98]. Studies carried out on two maize hybrids, one tolerant of (DKB 390) and the other sensitive to (BRS 1030) drought, showed that after foliar application of chitosan, higher photosynthesis was present in DKB 390 [99]. Moreover, research conducted on wheat (*Triticum aestivum* L. Jimai 22) also showed that the application of chitooligomers can improve photosynthesis [53]. It was also found that chitosan hexamers with different degrees of acetylation increase the photosynthetic rate and stomatal conductance of wheat seedlings [54]. Furthermore, chitosan showed a positive effect on photosynthesis in rice also under difficult conditions of elevated ozone concentration [87].

Under water deficit conditions on maize, studies were carried out on the effects of chitosan and two of its derivatives (*N*-succinylchitosan and N, O- dicarboxymethylated chitosan). Measurements conducted on leaves showed, on the first day after application, a decrease in carboxylation efficiency (P_N_/Ci) in comparison to control tests (both irrigated and stressed). However, after rehydration, the values in all treatments increased and were higher than the irrigated sample, in contrast to stomatal conductance (gs) and intercellular CO_2_ concentration (Ci), whose values decreased [96]. Scientists highlight the importance of chitosan in regulating the carbon and nitrogen metabolism in wheat [100]. Also, the potential of chitosan to regulate the carbon metabolism in rice was noted [79]. In a study on maize, foliar application of chitosan 140 mg L^−1^ was found to cause a decrease in gs. Thus it acts as an antiperspirant, without negatively affecting the P_N_ and the Ci [101].

## 7. Water-Use Efficiency (WUE)

There are two definitions of water-use efficiency (WUE). The first says that it is the ratio of photosynthesis to transpiration, also referred to in the literature as transpiration efficiency. The second describes it as the amount of carbon assimilated as biomass, or grain produced per unit of water consumed by the crop. For now, one of the key questions is how climate change (precipitation, temperature, and carbon dioxide) will affect plants and their WUE [102,103,104,105]. Therefore, the effect of antitranspirant use on this parameter is also important and of interest.

Studies carried out on wheat have shown that, after exogenous use of chitosan, WUE values improve. Furthermore, for plants treated with chitosan under moderate and severe drought stress, an increase in WUE was observed compared to untreated wheat and under normal conditions [26]. Also under salt stress conditions, it was shown that WUE was higher for wheat treated with chitosan compared to a control sample [43]. The influence of chitosan and its derivatives (*N*-succinylchitosan and N, O-dicarboxymethylated chitosan) under water stress conditions on maize was measured. Results showed that the WUE is higher for N, O-dicarboxymethyl and chitosan on both the first day of stress and after rehydration compared to two control samples (one irrigated and one stressed), in contrast to the values measured in the middle of the study duration, i.e., 15 days of stress, where results were lower [96].

Water-use efficiency was not a frequently analysed parameter in research on the chitosan effect on cereals. It is therefore difficult to draw universal conclusions. There are only a few studies that have taken this parameter into account. This should be changed, as WUE has a significant role in the adaptation and productivity of plants in water-limited areas. This concerns both the present as well as future climate change [106].

## 8. Relative Water Content (RWC)

The monitoring of water levels in plants is extremely important due to climate change and the intensification of droughts. One of the parameters used for this purpose is relative water content (RWC). It is responsible for biological water activity in plant tissues. In the assessment of the water balance of plants, RWC is one of the most important indices. Furthermore, RWC is a simple tool for farmers to monitor the water status of a plant to improve production [107]. Studies on the application of chitosan nanoparticles loaded with nitrogen, phosphorus, and potassium (NPK) on wheat showed an increased water content [39]. Also, foliar application of different concentrations of chitosan nanoparticles contributed to an increase in RWC for wheat. This is most likely due to a lower transpiration rate. The highest result of RWC was achieved at a level of 90 ppm of the substance [51]. Similar results, and the greatest improvement for the 90 ppm dose were also achieved with foliar application to barley (*Hordeum vulgare* L.) [52]. Furthermore, under reduced irrigation conditions for this plant, chitosan caused an increase in RWC [85]. In measurements on maize, RWC in plants treated with chitosan 140 mg L^−1^ for 15 days of drought conditions remained at the same level as in irrigated plants. At lower chitosan concentrations, a reduction in RWC during drought was recorded compared to well-watered plants [101]. The positive effect of chitosan on increasing RWC under drought conditions was also observed in experiments on rice [84]. Higher RWC values after chitosan treatment under drought stress were also observed in pearl millet [98]. Therefore it can be concluded that this treatment mitigated the impact of drought on the decrease in relative water content. In general, the application of chitosan and nanoparticles had a positive effect on RWC. More importantly, it also improved this parameter under drought conditions for major cereals such as wheat, maize, and rice. This is extremely important from the point of view of climate change, the increasing frequency of drought periods, and maintaining global food security.

## 9. Pathogen and Disease Control

The research carried out concerning the effect of chitosan on cereal diseases has been extensive. In this review, many papers were found on the impact of this product on different types of *Fusarium*. Studies on wheat and durum wheat (*Triticum durum*) have shown that chitosan is an effective tool to reduce the damage caused by the fungal pathogen *Fusarium graminearum* [108,109]. Scientists observed a decrease in the growth of *F. graminearum* at the lowest tested dose of chitosan 0.5 mg/g [110]. Research has also been conducted on durum wheat on the effect of hydrochloride chitosan. The results showed a decrease in the growth of *F. graminearum* [55]. Furthermore, it has been proven that in the case of wheat and barley, chitosan and isolates of *Pseudomonas spp*. cause a decrease in Fusarium seedling blight [111]. Also, promising effects were obtained when chlorophyllin-chitosan complex (Chl-CHS) was applied to wheat. Furthermore, in the case of this compound, no simultaneous reduction in growth and chlorophyll parameters was observed [112]. Also, research was conducted using a combination of chitosan and a plant biostimulant (liquid seaweed extract) prepared from a brown macroalga *Ascophyllum nodosum*. After using this combination, measurements on wheat leaves showed a significant decrease in the area infected by the *F. graminearum* pathogen [113]. The fact that chitosan as well as chitosan nanoparticles can control the disease *Fusarium* head blight, caused by fungi from the genus *Fusarium*, is also confirmed by other studies [114,115,116]. These results are very important, because *Fusarium* head blight disease is one of the important challenges for wheat cultivation.

The impact of chitosan on other diseases has also been studied. In other research on wheat, it was found that this substance suppresses *Septoria* leaf blotch disease [117]. Beneficial results were also obtained with chitosan hydrolyzate, which contained low-molecular-weight chitosan and its oligomer. It has been shown that a hydrolyzate concentration of 200 μg/mL causes total inhibition of *Septoria* leaf blotch of wheat [118]. Furthermore, measurements were also carried out on wheat using chitosan and its derivatives (Chit-V) as well as vanillin-modified chitosan. All these treatments decreased the leaf area infected by the phytopathogen *Cochliobolus sativus.* These substances were therefore found to induce resistance in wheat to *C. sativus*, which causes dark-brown blotch disease [119]. Moreover, the multifunctional complex ’Vitaplan CL + 0.1% Chitosan’ proved to be even more effective in the fight against wheat diseases. The study showed that its use decreased plant damage caused by brown rust by 20.6%. Also, it reduced the development of root rot, spots with powdery mildew, and the number of yellow rust strips. It is therefore an effective tool for decreasing disease incidence [41]. In the case of root rot, powdery mildew and leaf rust, studies have shown that other multifunctional biologics with chitosan also have the potential to defend wheat against this disease [42,120]. However, the results obtained with the application of ChitoPlant (Chitosan 99.9%) on the Edvins winter wheat variety are not conclusive. In one year of the study, this treatment was not effective against yellow rust, while in the following year an increase in effectiveness was observed. The authors state that this may be due to the different application timing and stress the need for further research [121]. It was also noted that chitosan reduced the percentage of wheat leaves infected by dark brown spot and also brown rust. In the control sample, leaf infection was 100%, while with chitosan it was only 25%. Even better results were obtained after treatment with chitosan in combination with salicylic acid. Leaf infection by dark brown spot was reduced to 20% and in the case of brown rust to 10% [122].

Furthermore, studies on maize showed that foliar application of chitosan reduced not only leaf spot disease, but also gray leaf blight, late wilt, and ear rot [123]. It was also found that chitosan had a positive effect on reducing leaf blight disease in this plant [32]. In addition, the potential of chitosan in fumonisin production by *Fusarium verticillioides* and *Fusarium proliferatum* has been investigated on maize samples. A decrease in growth of these *Fusarium* species was observed, using chitosan and water activity (aW) [124]. It also points to the huge potential of non-toxic chitosan to replace conventional antifungal agents [55]. A study on maize also found that chitosan in combination with *Cuscuta pedicellata* extract has the potential to be protective against *Fusarium oxysporum* [125]. The potential to increase resistance to *Fusarium oxysporum* was also observed in studies on rice. Chitosan oligosaccharide treatment was shown to reduce the incidence of seedling blight [126].

Studies also have been carried out on the effect of chitosan on rice crops (*Oryza sativa* L.) and their defensive reactions to stress and on the production of anti-fungal phytoalexins [127]. It has been proven that if rice leaves contain high levels of fibre, soaking the seeds in chitosan increases the potential for disease control and insect infection [49]. Measurements were also carried out on rice after the application of lanthanum-modified chitosan oligosaccharide nanoparticles (Cos-La). It was demonstrated that Cos-La induced resistance to rice blast [82]. Also, the use of chitosan guar nanoparticles may be effective in controlling rice blast, as well as blight disease [128]. Moreover, it has been observed that the percentage of rice attacked by brown planthopper after application of chitosan is reduced. This is very important, because this insect is one of the causes of crop losses in rice yields [50]. Also, chitosan-magnesium (CS-Mg) nanocomposite have proved to be effective in inhibiting the growth of two rice pathogens *Acidovorax oryzae* and *Rhizoctonia solani* [129]. For *Rhizoctonia solani*, chitosan nanoparticles without additives were also successful in suppressing this pathogen [130]. Also, after the use of chitosan, inhibition of mycelium development, as well as the occurrence of the disease caused by this pathogen was observed [131]. These results suggest the potential of chitosan and its derivatives in combating rice sheath blight. One study even says that chitosan reduced the severity of this disease by up to 89% [132]. Moreover, chitosan applied to rice was found to enhance defense responses against other pathogens. The potential of 0.25% chitosan to control *Aphelenchoides besseyi*, the rice pathogen that causes white tip disease, has also been noted [133]. Moreover, the effectiveness of defense against rice blast pathogen *Magnaporthe grisea* has been proven. It has been observed that chitosan with low molecular weight contributes to higher resistance than with high molecular weight [134]. Studies have also been conducted on the effect of chitosan-silver nanoparticles combined with trihexad on rice blast caused by fungus *Pyricularia oryzae*. The antifungal potential of this chemical combination has been indicated [135]. It has also been found that *N,O*-acylchitosan has the potential to control *Botrytis cinerea* gray mold and *Pyricularia oryza* blast mould on rice. Furthermore, this derivative has shown better results than chitosan treatment alone [136]. The potential to control *Pyricularia oryzae* has also been demonstrated with the treatment of chitosan nanoparticles loaded with protocatechuic acid [137]. All these results suggest that chitosan and its variants can help to effectively combat rice blast. This is extremely important, as this disease contributes to significant crop damage. Other diseases that reduce the yield of rice are bacterial leaf blight and leaf streak. Studies have shown that chitosan has an antibacterial effect and is capable of preventing this disease, too [138]. Furthermore, biosynthesised chitosan nanoparticles have also shown inhibitory effects on the growth of *Xanthomonas oryzae* pv. *oryzae (Xoo)*, a pathogen that causes bacterial leaf blight of rice [139]. Research has also been conducted on the use of COS-OGA, which are formulations of chitosan oligomers (COS) and pectin-derived oligogalacturonides (OGA). It was shown that COS-OGA can control root-knot nematode caused by *Meloidogyne graminicola* on rice [140].

Tests carried out in both greenhouse and field conditions on pearl millet showed that chitosan seed priming protects crops from downy mildew. Furthermore, the effect of chitosan on the regulation of resistance gene analogue RGPM213 to infection by downy mildew pathogen was also investigated in pearl millet [141]. The use of this substance causes systemic and durable resistance to this type of infection [58,142,143]. This has also been confirmed by later studies of seed treatment with nanochitosan particles [59]. The results of these research works are extremely significant, because this is the most devastating disease with pearl millet. This plant is resistant to drought and heat, and is grown mainly in Asia and Sub-Saharan Africa, where it is one of the basic foods for poor people [144,145]. The second plant in this group, on which the influence of nanochitosan particles was studied, was finger millet. The measurements showed that they inhibit the development of *Pyricularia grisea,* which causes blast disease. Moreover, the use of this substance resulted in a lower frequency of the disease [45]. It has also been shown that the application of copper-chitosan nanoparticles (CuChNp) on finger millet causes protection against blast disease [60]. The same conclusions were obtained for the treatment of oligochitosan on rice [80]. Moreover, the potential after treating seeds with chitosan and hydrolyzed chitosan for defense reactions to *Pyricularia grisea* (Cooke) Sacc. was also shown by research on the same plant [146]. Similar conclusions were reached for chitosan nanoparticles [147]. All of the above-mentioned results indicate that both chitosan and its varieties are effective tools in the fight against blast disease in cereal crops.

The potential of this product to control powdery mildew on barley (*Hordeum vulgare* L.) plants was also analysed and it was noted that it could slow the spread of the fungus [148]. Experiments with the use of chitosan on winter wheat (*Triticum aestivum* L.) indicate that there is a need for further research into the impact of this measure on pink snow mould (*Microdochium nivale*) [149]. Also, it has been shown that under the abiotic stress of maize, the use of chitosan reduces the development of the fungi *A. flavus* and *F. moniliforme* [150]. Subsequent studies on maize and sorghum confirmed that the application of chitosan had a fungistatic effect against *A. flavus* and a fungicidal effect against *Rhyzopus* spp. [69]. In studies involving the use of Cu-chitosan nanoparticles on maize, it has been shown that this substance can act as an antifungal preparation for *Curvulari*a leaf spot (CLS) disease [35]. It has been proven that Zn-chitosan nanoparticles (NPs) also have such properties and cause higher mycelial growth inhibition [37]. Furthermore, it has been shown that Cu-chitosan nanocomposites are an effective antifungal agent against post-flowering stalk rot disease on maize [151]. Research on the same plant using salicylic acid-chitosan nanoparticles (SA-CS NPs) has also shown positive results for controlling this disease [77]. Scientists also note the need to consider chitooligosaccharides with different degrees of acetylation in the context of the use of protective preparations containing chitosan and the regulations for their use [152]. Therefore, research has also been carried out into the effect of chitooligosaccharides as well as chitooligosaccharides with different degrees of acetylation on reducing root rot in wheat and changes in the level of gene expression of pathogens [153,154]. It has been shown that the use of this derivative of chitosan stimulates defensive reactions to the necrotrophic fungus *Bipolaris sorokiniana*, which is responsible for the rot [153]. It has also been proven that this treatment strengthens the resistance of wheat calli to the bunt fungal pathogen *Tilletia caries* (DC) Tul. [155]. However, the researchers still stress the need for further studies on chitosan to increase data on its impact on crop diseases [156].

Based on the above overview, chitosan is highly effective in reducing the spread of cereal diseases. Its application is very broad. It has a beneficial influence, regardless of the crop type. This is particularly important for cereals, which are largely responsible for feeding the world’s population. Chitosan and its nanoparticles have proven to be effective against rice diseases such as blast and sheath blight. These diseases are the main factors preventing stable rice production in the world [157]. However, the researchers also emphasise that despite promising results in disease control, chitosan nanoparticles have not yet found widespread application in rice cultivation [130]. In the case of finger millet, chitosan showed effective protection against not only blast disease but also downy mildew. It also affected many other pathogens occurring in barley or maize crops. Moreover, it reduces the most important wheat diseases such as rusts, blotches, and head blight. This is particularly significant considering that fungal diseases can cause yield losses of 15–20% per year [158]. Furthermore, cereal diseases also affect harvest quality and safety in terms of toxins [159]. Based on this review, it can be concluded that cereal disease damage can be reduced by the widespread application of chitosan during cultivation.

## 10. State of Knowledge and Research Trends

Since 2000, 188 original scientific articles on the use of chitosan, its derivatives, nanoparticles, or chitosan fertilizers on cereals have been published. All papers considered for this article were found in the Web of Science, Scopus, or Google Scholar databases. This review includes not only articles describing the field application of chitosan on cereals but also laboratory studies covering the effects of the antitranspirant on plant traits and disease reduction. All the papers found in the databases during this review are listed in Table 1. Most of the studies focused on the use of chitosan, chitooligosaccharides (COS), or nanoparticles. Among nanoparticles, Zn-chitosan nanoparticles, Cu-chitosan nanoparticles, chitosan-silver nanoparticles, and also chitosan-La nanoparticles were found. Applications also included irradiated chitosan, chitosan hydrochloride, chitosan nanoemulsion, or chitosan fertilizers.

**Figure 1:**
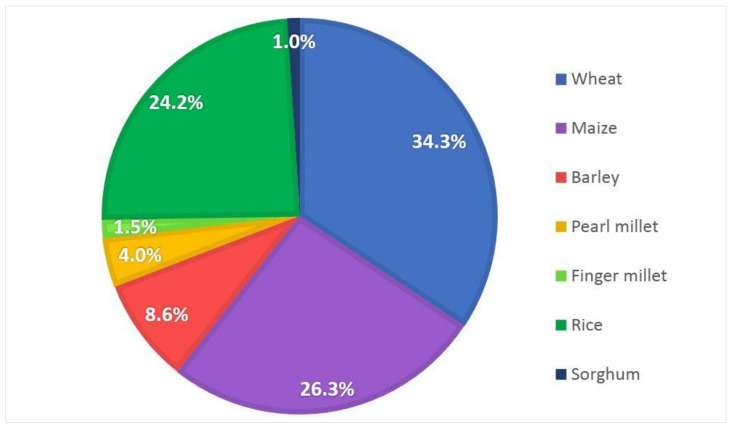
Share of each cereal in original scientific papers on the use of chitosan.

This review showed that wheat is the most popular cereal in the study, which accounts for 34.3% of all measurements (Figure 1). The second most frequent cereal in published papers is maize (26.3%) and the third is rice (24.2%). The percentage of research on barley is 8.6% and pearl millet only 4.0%. Studies on the use of chitosan on sorghum or finger millet are very rare. Their percentage contribution to the total is 1.0% and 1.5% respectively. This review indicates the need for more measurements on these crops to better understand the impact of chitosan on cereals. Furthermore, excluding studies on grain storage and further food processing, no publications on the use of chitosan on either oats or rye as plants have been found since 2000. This indicates a clear tendency for researchers to consider the most popular cereals and omit from their studies those with smaller cultivated areas worldwide. However, it should not be forgotten that to better identify the effect of chitosan, less common plants belonging to the cereal group should be considered.

**Figure 2:**
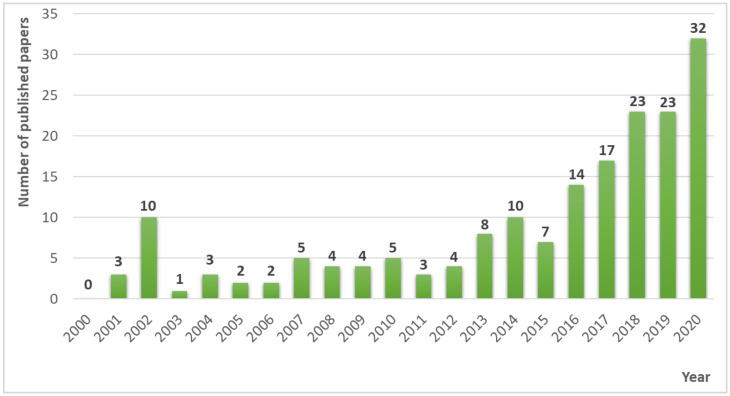
Number of original scientific articles on the use of chitosan and its derivatives and nanoparticles on cereals published between 2000 and 2020.

This does not change the fact that the enormous potential of the application of chitosan, its derivatives, or nanoparticles on cereals is being increasingly recognized worldwide. Consequently, the application of chitosan-based products in agriculture is growing in popularity. In China alone, there are more than 50 different chitosan-based bioproducts with officially issued certificates [100]. Scientific research is also increasingly taking into account the use of this product. This has been particularly evident in recent years. Since 2016, the number of research papers focusing on the use of chitosan and its derivatives or nanoparticles on cereals has increased dramatically (Figure 2).

In 2020, a total of 32 articles were published, which is four times more than in 2013. Moreover, in 2000 there was not a single paper on this topic. The noticeable increasing trend of interest in this topic is very positive, because antitranspirants including chitosan can be an excellent tool for adaptation of cereals to climate change due to their high-stress tolerance. Also, the measurements and cell analyses carried out indicate that chitosan is not toxic.

It should be emphasised that this also includes high concentrations of this substance [71]. It has also been noted that the application of chitosan is environmentally friendly and reduces production costs [50]. The significant increase in scientific research in this area allows valid and accurate conclusions to be drawn and will contribute to a faster implementation of the widespread use of chitosan on cereals.

## 11. Conclusions

Based on this review, a marked increase in interest regarding the use of chitosan on cereals has been noted in recent years. Since 2000, 188 papers have been published on the use of chitosan and its derivatives and nanoparticles on cereals. The most popular were articles on wheat, maize, and rice. The need to expand the number of measurements on less popular cereals such as finger millet, pearl millet rye, oats, and sorghum has also been noted. Thanks to increased research activity in recent years, the state of knowledge on the effects of antitranspirants has improved considerably. It is clear from the review that chitosan, as well as its derivatives and nanoparticles, are non-toxic and have positive effects on cereals. Their application contributes to increased yields, not only under normal conditions but also during drought stress, salt, or low temperatures. Most studies also show positive effects on certain growth parameters. However, a more detailed analysis, taking into account the application method, concentration, and individual factors during the measurements is needed to formulate specific conclusions on cereal. Moreover, there is a tendency to alleviate the effects of stress and to improve growth parameters under unfavourable conditions. Regardless of the type of cereal, this antitranspirant also has a positive impact on chlorophyll content. Furthermore, this review has shown that chitosan is highly effective against pathogens and cereal diseases such as fusarium seedling blight, blast disease, septoria leaf blotch, downy mildew, and many others. It significantly reduces damage caused by the most threatening diseases to maize, wheat, rice, barley, and millet. Its application is effective regardless of the type of cereal and, more importantly, it covers a very wide range of pathogens. This demonstrates the enormous application value of chitosan in agriculture. Effective control of diseases, which is currently one of the biggest challenges in cereal cultivation, will significantly limit harvest losses. Chitosan, with its environmentally friendly composition and high effectiveness, can be an excellent alternative to synthetic fertilizers, pesticides and chemicals for disease control. Also, treating cereals with this product will reduce the negative effects of drought, salt, and low temperature stress on crops and contribute to higher yields. This shows the enormous potential of chitosan as a universal product for improving cereal yields regardless of environmental conditions. 

## Figures and Tables

**Table 1:** Summary of papers on chitosan, its derivatives and cereals published since 2000.

Chitosan Type	Cereal	References
Chitosan	Barley	[30,31,85,111,114,148,160,161,162,163]
	Maize	[29,32,33,47,61,62,69,70,71,72,73,74,91,95,96,99,101,110,123,124,125,150,164,165,166,167]
	Pearl millet	[141,142,143,168]
	Rice	[48,49,50,78,79,84,86,87,127,129,131,132,133,134,138,146,161,169,170,171,172,173,174,175,176,177,178]
	Sorghum	[69,179]
	Wheat	[25,26,27,28,43,100,108,109,110,111,113,114,115,116,117,119,121,122,149,161,167,169,180,181,182,183,184,185,186]
Chitooligosaccharide (COS)/ chitosan oligomer/ chitooligomer	Barley	[187,188]
	Rice	[80,81,89,126,189,190,191]
	Wheat	[40,53,88,90,100,152,153,154,155,185,192,193,194]
Chitosan nanoparticles	Barley	[52,57]
	Finger millet	[46]
	Pearl millet	[45,59]
	Rice	[67,130,139]
	Wheat	[51,56,116]
Cu-chitosan nanoparticles	Finger millet	[60]
	Maize	[35,75,76,151]
Zn-chitosan nanoparticles	Maize	[37]
	Wheat	[195,196,197,198]
Chitosan nanoparticles loaded with nitrogen, phosphorus and potassium (NPK)	Wheat	[38,39]
Irradiated chitosan	Barley	[199,200]
*N*-succinylchitosan and *N,O*-dicarboxy-methylated chitosan	Maize	[33,96]
*N,O*-acylchitosan (NOAC)	Rice	[136]
Oligochitosan	Wheat	[201]
Deacetylated chitosan	Wheat	[202]
Vanillin-modified chitosan	Wheat	[119]
Chitosan nanoemulsion	Pearl millet	[98]
	Wheat	[203]
Chitosan hydrochloride	Wheat	[55,204]
Chitosan-La nanoparticles, lanthanum-modified chitosan oligosaccharide nanoparticles (Cos-La)	Rice	[82]
Chitosan silver-nanoparticles	Rice	[135]
Moringa with chitosan and iron (MHCFe) nanoparticles	Maize	[68]
Chitosan nanoparticles containing *S*-nitrosomercaptosuccinic acid (*S*-nitroso-MSA)	Maize	[97]
Salicylic acid-chitosan nanoparticles	Maize	[77]
Sepiolite-chitosan nanocomposites	Maize	[205]
Chitosan nanoparticles loaded with *N*-acetylcysteine	Wheat	[44]
Chitosan nanoparticles loaded with protocatechuic acid (PCA)	Rice	[137]
Chitosan guar nanoparticle	Rice	[128]
Chitosan nanoparticles encapsulated with *Cymbopogon martinii* essential oil	Maize	[206]
Aqueous commercial formulation containing 4% chitosan name Elexa	Pearl millet	[58]
Chitosan and PUSA hydrogel	Wheat	[28,186]
Chitosan and plant biostimulant made from *A. nodosum* (liquid seaweed extract or LSE)	Wheat	[113]
Chitosan and hydrogel	Wheat	[27]
Chlorophyllin-chitosan complex (Chl-CHS)	Wheat	[112]
Chitin and chitosan hexamers, homogeneous chitosan hexamers [(GlcN)6]	Wheat	[54]
Vitaplan, CL + chitosan	Wheat	[41,42,120]
Chitosan hydrolyzate	Wheat	[118]
Formulations of chitosan oligomers (COS) and pectin-derived oligogalacturonides (OGA), COS-OGA	Rice	[140]
Modified chitosan (MCTS), gibberellin, glutamic acid, sodium	Rice	[207]
Modified chitosan (MCTS), gibberellin, glutamic acid, sodium α-naphthalene acetic acid, sodium bentonite, polyvinyl acetate (PVAc), potato dextrose agar (PDA), emulsifier (OP-10), ethylene glycol (EG), polyethylene glycol 1000 (PEG-1000)	Maize, Barley, Wheat	[208]
Chitosan/sodium alginate hydrogel rings loaded with chlorantraniliprole (CLAP)	Maize	[209]
Chitosan/tripolyphosphate nanoparticles loaded with paraquat herbicide	Maize	[210]
Gel of chitosan and ZnO nanoparticles, CH-ZnO4 chitosan-ZnO nanoparticles	Maize	[211]
Paraquat-loaded pectin/chitosan/tripolyphosphate nanoparticles	Maize	[212]
Carboxylated graphene oxide-chitosan spheres	Wheat	[213]
Chtiosan/nanochitosan with plant growth- promoting rhizobacteria (PGPR)	Pearl millet	[214]
	Maize	[34,66]
	Rice	[215]
Fertilizer/nanofertilizer with chitosan	Maize	[36,63,64,65,216]
	Wheat	[217]
Quitomax^®^ bioproduct based on chitosan	Rice	[218]

## Data Availability

Not applicable.
